# Effectiveness of a Virtual Reality Open-Air Bath Program in Reducing Loneliness and Improving Brain Function for Dementia Prevention in Older Adults: Protocol for a Prospective Randomized Crossover Study

**DOI:** 10.2196/57101

**Published:** 2024-08-01

**Authors:** Ayu Imai, Teruyuki Matsuoka, Chikara Nakayama, Nana Hashimoto, Mutsuo Sano, Jin Narumoto

**Affiliations:** 1 Department of Psychiatry Graduate School of Medical Science Kyoto Prefectural University of Medicine Kyoto Japan; 2 Department of Psychiatry National Hospital Organization Maizuru Medical Center Maizuru Japan; 3 Department of Information Science and Technology Osaka Institute of Technology Osaka Japan

**Keywords:** loneliness, virtual reality, VR, Alzheimer disease, predementia, intervention, subjective cognitive decline, mild cognitive impairment, dementia, older adult, geriatric, depression, cognitive impairments

## Abstract

**Background:**

Older adults often face loneliness due to chronic illness or loss of close ones, a situation worsened by the COVID-19 pandemic. Increased loneliness heightens the risk of diseases, especially dementia, necessitating urgent action.

**Objective:**

This study aims to assess the impact of a virtual reality (VR)–based open-air bath program on depression and loneliness in older individuals with subjective cognitive decline/mild cognitive impairment attending the Dementia Medical Center in Kyoto, Japan. We further aim to evaluate the feasibility of the program (participant recruitment and adherence) and to measure program enjoyment and satisfaction.

**Methods:**

The study design is a crossover trial with a 1:1 ratio, wherein 12 participants will be randomly assigned to groups 1 and 2, with group 2 serving as a waitlist control and group 1 receiving the VR program from the onset for 6 months; the VR program will be conducted 6 times (monthly). Program completion for group 1 will be followed by an observation period from months 7 to 12. Group 2 will participate in the VR program from months 7 to 12, with an observation period from months 1 to 6. Cognitive tests, psychiatric assessments, and the University of California, Los Angeles Loneliness Scale will be conducted before the study, at 6 months, and at 12 months. Results will be analyzed using repeated-measures ANOVA. Head magnetic resonance imaging and single-photon emission computed tomography scans will be performed before and after the VR program to evaluate changes and effects on brain regions.

**Results:**

Recruitment began in September 2023 and data collection is expected to be completed by March 2025. Complete study results will be published by September 2025.

**Conclusions:**

This study examines the preliminary effects of VR on loneliness in older adults with predementia through open-air bath simulations. VR experiences could benefit this population, particularly those with limited outdoor activities. Quantifying VR’s impact will aid in determining the size for a larger clinical trial. Qualitative results will inform participation mechanisms and guide the implementation and design of future trials.

**Trial Registration:**

University hospital Medical Information Network UMIN000052667; https://tinyurl.com/3yaccay5

**International Registered Report Identifier (IRRID):**

DERR1-10.2196/57101

## Introduction

### Background

In recent years, we have experienced social isolation on an unprecedented scale, partly due to the spread of infectious diseases. The Japanese government has addressed the issue of isolation and loneliness by establishing a Minister of State for Isolation and Loneliness in 2021. This health issue is attracting global attention, with the World Health Organization also emphasizing the importance of measures to combat loneliness [1]. Feeling lonely and socially isolated can seriously impact physical and mental health. A review distinguished between loneliness and isolation, noting that loneliness is subjective and psychological, while isolation is objective and social. However, social isolation does not necessarily imply loneliness, and loneliness is a stronger predictor of mental and physical health status than social isolation [2]. Loneliness is an independent risk factor for depression, even after accounting for covariates such as demographic characteristics, marital status, social isolation, and psychosocial risk factors [3]. The health burden of loneliness is extensive, including increased mortality from hypertension and cardiovascular disease, heightened risk of diabetes and immune system dysfunction, and increased risk of suicide [4-6]. In a recent meta-analysis, loneliness was identified as a risk factor for coronary heart disease and stroke [7]. The odds ratio of increased mortality due to loneliness is approximately 1.5, which is comparable to smoking and heavy drinking and exceeds the effects of other risk factors such as obesity and physical inactivity [8]. Lonely individuals often have poor mental health, which is reported to increase the risk of dementia as well as depression and other mental illnesses.

### Impact of Loneliness on Dementia

Older adults who are lonely are 1.64 times more likely to develop dementia than those who are not [9]. Furthermore, loneliness can exacerbate the behavioral and psychological symptoms of dementia [10]. As one ages, the likelihood of experiencing loneliness increases, with 30%-40% of older adults reporting feelings of loneliness [11]. The growing global population of people with dementia and the increasing costs of medical and nursing care are placing a significant burden on younger generations [12,13]. Therefore, preventing loneliness and isolation among older adults is a pressing worldwide issue. Although the exact mechanism by which loneliness increases the risk of dementia is not fully understood, it has been linked to amyloid load and tau pathology, as well as decreased gray matter volume in the thalamus and parahippocampal gyrus, potentially accelerating the pathology of Alzheimer disease due to the increased strain on the brain [14-16].

### Interventions to Reduce Loneliness

Reducing social isolation and loneliness can help prevent dementia [17]. Reviews of interventions for social isolation and loneliness have shown that participation in meaningful and satisfying group activities is effective in reducing loneliness [18]. However, opportunities for such activities are declining due to restrictions on outings and social distancing considerations during infectious disease outbreaks [19]. Even when infectious diseases are at an all-time low, many older adults find it challenging to go outside due to underlying medical conditions. Group activities using virtual reality (VR) can be enjoyed by these individuals as they can participate from the comfort of their own homes or facilities. The immersive experience provided by the headset can increase motivation to participate compared to mere video images.

### VR Program to Reduce Dementia Risk

Subjective cognitive decline (SCD) and mild cognitive impairment (MCI) are known as a preliminary stage or risk factor for dementia [20,21]. Intervening with VR programs for people with these conditions may reduce their sense of isolation and decrease the risk of dementia. Moreover, such interventions may not only improve mental functions, such as creating enjoyment in the lives of older adults and increasing their motivation to live, but may also help prevent physical diseases such as cardiovascular diseases and diabetes. Although various studies have been conducted to test methods of reducing loneliness among older adults [22], there are no studies examining the effects of interventions on cognitive function, mental status, and the risk of dementia, and there is no precedent for VR-based initiatives. Furthermore, although VR for rehabilitation and training of cognitive functions in patients with MCI has been developed and shown to be useful for improving general cognitive functions, especially executive functions [23,24], there is no precedent for a program aimed specifically at improving loneliness.

Therefore, we hypothesize that meaningful activities using VR programs could reduce social isolation and loneliness among older people, thereby reducing the risk of dementia. This study has two objectives. The first is to examine the effects of a VR-based open-air bath experience program on depression and loneliness among older adults with SCD and MCI attending our Medical Center for Dementia Diseases. The second objective is to examine feasibility indicators such as recruitment and adherence status, program enjoyment, and satisfaction. In this study, participants will be randomly assigned to 2 groups: a VR group (group 1) and a waitlist control group (group 2). The design of the study is a crossover, superiority trial with an allocation ratio of 1:1.

## Methods

### Study Design

This is an intervention and prospective study with minor invasiveness using VR and the protocol is developed according to the SPIRIT (Standard Protocol Items: Recommendations for Interventional Trials) guidelines [25] (Multimedia Appendix 1). Two groups of 6 people each, totaling 12 participants, will be recruited from a group of older adults who visited the Medical Center for Dementia Diseases at Kyoto Prefectural University of Medicine and were diagnosed with SCD or MCI. This number meets the minimum recommendations of a feasibility study considering the sample size for a large clinical trial [26]. Participants will visit the outpatient clinic once a month for a total of 12 times for standard outpatient care, including examination, evaluation, and prescribing. Although this is the standard frequency of visits at our center, monthly visits are expected to improve adherence to this protocol and prevent dropout.

During this 12-month period, changes in pharmacotherapy will be minimized but made when necessary. During the period of the VR program, participants will receive the VR program in addition to outpatient care. In the observation period, they will chat with the same members for the same amount of time in addition to outpatient care.

The allocation method will be simple randomization with a 1:1 ratio to either group 1 or group 2 based on computer-generated random numbers. Due to the nature of the intervention, double-blinding is challenging. Therefore, to minimize measurement bias, only the data collectors and analysts will be blinded. An investigator not involved in the study will perform the allocation and only the facilitator will be informed of each group’s allocation. Group 1 will participate in the VR program once a month from the start to 6 months, with an observation period from 7 to 12 months. Group 2 will participate in the VR program monthly from 7 to 12 months, with an observation period from 1 to 6 months from the start. Participation in the VR program is planned to involve 6 sessions in total (once a month).

**Figure 1:**
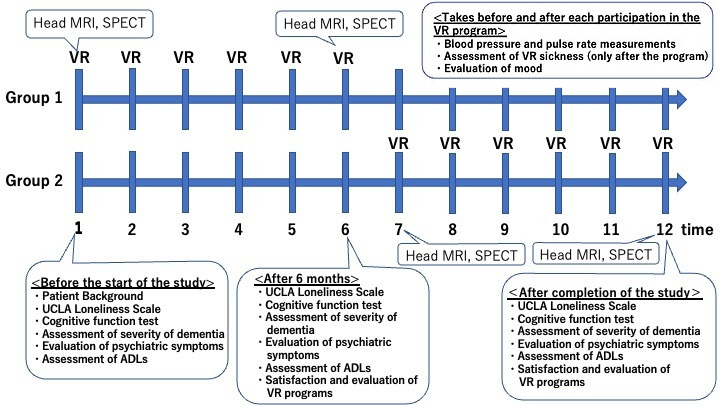
Schedule and assessment timeline for the virtual reality (VR)–based open-air bath experience program. ADL: activities of daily living; MRI: magnetic resonance imaging; SPECT: single-photon emission computed tomography; UCLA: University of California, Los Angeles.

The loneliness scale, stress index, various cognitive function tests, and psychiatric symptom evaluation will be conducted before, at 6 months, and 12 months after participation to observe changes due to participation in the VR program. The detailed study procedure is depicted in Figure 1. These evaluations will be performed by a psychiatrist or psychologist skilled in geriatric psychiatry. No statistical analysis will be performed for adverse events or impressions. This study has been registered in the University Hospital Medical Information Network (UMIN) (UMIN000052667).

### Ethical Considerations

The protocol and informed consent were approved by the Ethical Review Board of Kyoto Prefectural University of Medicine on October 31, 2023 (ERB-C-2978). Study participants will be informed in writing and consent will also be obtained in writing (Multimedia Appendix 2). Consent can be revoked at any time without any disadvantage to the participant, in which case the data will be discarded except for those necessary for medical treatment. Any important modifications to the protocol will be promptly submitted to the institutional review board or UMIN for approval, and research participants will be informed by telephone to obtain their consent to continue participation in the study. Although the VR may cause VR sickness, this is considered a minor invasion and the collection of information is not invasive. The examination items consist of items used in routine medical care and are not invasive beyond routine clinical practice. Therefore, no compensation will be provided because no health hazard is expected for the research participants as a result of this study.

Data, anonymized and managed by assigned numbers, will be entered into an Excel table and stored securely. Access to the intermediate and final datasets will be limited to the principal investigator JN and coinvestigators AI, TM, CN, and NH. The data manager, who is not involved in this study, will oversee data handling. Adverse events or unexpected intervention effects will be documented and reported to the principal investigator. The research team will collectively decide on the continuation or discontinuation of the intervention upon receiving such reports. The principal investigator will make the final decision on study termination.

### Eligibility for Participation

Participants will be older adults aged ≥60 years diagnosed with SCD or MCI who visited the Medical Center for Dementia Diseases at Kyoto Prefectural University of Medicine Hospital between November 1, 2023, and March 31, 2025, and who can provide written consent to participate in the study. The Petersen criteria for MCI diagnosis [27] and the definition of SCD proposed by Jessen et al [28] (ie, a condition of ongoing subjective cognitive problems without dementia or MCI) will be used. Participants complaining of SCD but judged by expert psychiatrists to have normal cognitive function based on neuropsychological tests such as the Mini Mental State Examination (MMSE) [29], Alzheimer’s Disease Assessment Scale (ADAS) [30], Rivermead Behavioral Memory Test [31], and Clock Drawing Test [32] and brain imaging tests will be diagnosed as having SCD. The purpose of the study along with the benefits and disadvantages will be explained in writing, and only those who agree to participate will be included. Patients with a history of mental illness, head injury, or drug or alcohol abuse; those with significant visual or hearing impairment; and those unable to use both hands will be excluded.

### Procedure

The primary endpoint will be the score on the loneliness scale. The secondary endpoints include cognitive function tests (MMSE and ADAS); assessment of depression (Geriatric Depression Scale [GDS] [33]), dementia severity (Clinical Dementia Rating [CDR] [34]), and activities of daily living (ADLs; Instrumental Activities of Daily Living [IADL] and Physical Self-Maintenance Scale [PSMS] [35]); and the Mild Behavioral Impairment Checklist (MBI-C) [36,37]. All of the above assessments will be taken the day of consent. Patient background (age, gender, educational history, dominant hand, medical history, and presence of a roommate) will also be obtained via an interview conducted the same day.

Loneliness will be assessed using the revised University of California, Los Angeles (UCLA) Loneliness Scale [38,39]. This 20-item scale has scores ranging from 20 to 80, with higher scores indicating more severe loneliness. If a participant has already obtained a score on this scale in the past 3 months, that information from their medical record or other sources may be used. If fatigue or other complaints occur during these tests and all test items cannot be obtained on the same day, the schedule will be adjusted and the remaining test items will be performed within 1 month from the original test date.

The cognitive function and clinical rating scale assessments will be conducted by a data collection staff member through questionnaires and interviews at the psychiatric outpatient department of the hospital on weekdays between 9 AM and 5 PM. This staff member will be the psychologist who is usually in charge of cognitive function tests and interviews at the Medical Center for Dementia Diseases and is familiar with the methods used. The results will be explained to the patient if desired.

Immediately before and after implementation of the VR program, blood pressure and pulse rate measurements will be taken along with evaluation of mood state. Immediately after the program, an evaluation of VR sickness will be conducted. All participants will be evaluated for the loneliness scale, cognitive function test, psychiatric symptoms, dementia severity, and ADLs at the time group 1 completes the program (after 6 months) and when group 2 completes the program (after 12 months). Head magnetic resonance imaging (MRI) and single-photon emission computed tomography (SPECT) will be performed at the beginning and end of the VR program. Satisfaction and evaluation of this program will be assessed at the end of the VR program.

If VR sickness occurs more than expected, we will consider changing the program specifications. Furthermore, if a participant requests discontinuation or is unable to continue participation for some reason, the intervention will be stopped only for that patient. Participants who discontinue or withdraw from the intervention will have the reasons for discontinuation or withdrawal confirmed and recorded, and data collected up to that point will be used if approved. To prevent dropouts, the program will be scheduled every 4 weeks on Monday afternoons, and the schedule will be distributed to participants and their families in advance.

### Program Content

This 6-month program offers participants a 20-minute virtual open-air hot spring experience once a month. In this program, participants virtually visit hot spring resorts around the world through VR while enjoying a footbath with a bucket and bath salts, mimicking a real hot spring resort experience. The program features actual hot spring scenery and environmental sounds, with no actual people appearing in the program. The open-air bath simulation occurs in an outpatient consultation room and involves a facilitator and an attendant. Participants use an Oculus Quest 2 headset and immerse their feet in 40 °C water in a footbath bucket. During the 20-minute session, participants and the facilitator are encouraged to converse naturally, although no specific script is provided. The VR sessions are conducted in a medical examination room with appropriate infection prevention measures in place, including temperature checks, proper ventilation, mask-wearing, hand sanitization, and maintaining a distance of at least 1 meter. When the VR program is not in session, patients will engage in a 20-minute conversation with the facilitator and attendant once a month in addition to their regular medical examination.

### Analysis

As a preliminary study, this research focuses on estimating outcomes rather than determining validity. The findings will inform the sample size and design for larger studies. Statistical analysis will include descriptive statistics (mean, SD), effect sizes, and 95% CIs. Baseline characteristics such as age, gender, diagnosis, and cohabitation status will be compared for each group. The revised UCLA Loneliness Scale will serve as the primary endpoint, with assessments conducted at baseline, 6 months, and at the end of the study. Comparisons between the groups will be made using 2-way repeated-measures ANOVA. Secondary endpoints, including MMSE, ADAS, GDS, MBI-C, CDR, and PSMS scores, will undergo a similar analysis. Changes in brain volume and cerebral blood flow, as observed in head MRI images and SPECT scans, will be compared using a paired *t* test before and after the intervention with Statistical Parametric Mapping software. Multiple regression analysis will be used to explore the relationship between changes in loneliness scale scores and preintervention brain images to identify brain regions predicting improvements in loneliness. Additionally, changes in mood state and vital signs before and after the program will be compared using paired *t* tests. The degree of VR sickness and satisfaction with the program will be described in a report. 

Participant discontinuation or dropout cases will be excluded from the analysis, but their numbers will be clearly noted. Missing values will be deleted or imputed using appropriate methods.

## Results

This study was approved by the Ethics Review Committee of Kyoto Prefectural University of Medicine on October 31, 2023. The study was initiated on November 1, 2023, and the first participants were enrolled on December 18, 2023. Recruitment of the last participant is scheduled for March 31, 2025, and the study is projected to be complete by September 2025.

## Discussion

This study aims to explore the preliminary effects of a VR-based open-air bath experience program on loneliness in older adults at the predementia stage. Given the tendency of this population to limit outdoor activities due to infectious disease outbreaks and other factors, a VR-simulated outdoor bath experience could be highly beneficial and may help alleviate depressive symptoms and feelings of loneliness. The study will quantify the effects of the VR program on these factors and provide data that could inform the sample size for a larger-scale clinical trial. Additionally, the qualitative results will enhance our understanding of the behavioral mechanisms that promote participation and continued use, thereby informing the design and implementation procedures for future trials.

Loneliness in older adults is often the result of a combination of health and environmental factors. Health-related factors include chronic illness and communication difficulties [40] as well as cognitive decline impairing the ability to recall recent conversations or appointments [41]. Environmental factors contributing to loneliness include a lack of friends, moving to a nursing home, the bereavement of a loved one, and excessive time spent alone, all of which increase feelings of isolation from others [42,43]. Lonely people are at high risk for reduced life satisfaction, depression, low self-esteem, negative emotions, and impaired functioning in ADL, all of which can impair health and well-being [44,45].

Various interventions have been implemented to promote social connectedness and reduce loneliness in older adults. These include group activities such as cognitive enhancement workgroups and participation in adult day centers, one-on-one interventions such as befriending and animal-assisted therapy, internet access training, and service provision such as transportation and medical care [46-48]. Studies focusing on reducing loneliness have identified effective interventions such as those increasing social contact opportunities, enhancing social support, focusing on social skills, and addressing maladaptive social cognitions through methods including cognitive behavioral therapy [18,49]. However, these programs often have limitations such as difficulty with equipment-handling techniques and infectious diseases, along with challenges for older individuals who struggle to leave their homes. The VR program used in this study offers the advantage of easy participation by simply wearing a head-mounted display. The VR program can be used from any location with a network environment, including homes and facilities.

Notably, there have been no previous studies examining changes in cognitive function, brain blood flow, and brain structure as a result of interventions aimed at reducing loneliness. However, this study has certain limitations, including its small sample size. The findings will need validation in a larger clinical trial. Additionally, the requirement for participants to see the screen of a VR headset may not be suitable for those with visual impairments.

In conclusion, should the results indicate a benefit to the mental health of older adults in the predementia stage, this study may pave the way for innovative and scalable methods to enhance both the mental and physical health of this population group.

## Appendix

Multimedia Appendix 1 SPIRIT (Standard Protocol Items: Recommendations for Interventional Trials) checklist.

Multimedia Appendix 2 Consent form.

## Abbreviations

ADAS: Alzheimer’s Disease Assessment Scale
ADL: activity of daily living
CDR: Clinical Dementia Rating
GDS: Geriatric Depression Scale
IADL: Instrumental Activities of Daily Living
MBI-C: Mild Behavioral Impairment Checklist
MCI: mild cognitive impairment
MMSE: Mini Mental State Examination
MRI: magnetic resonance imaging
PSMS: Physical Self-Maintenance Scale
SCD: subjective cognitive decline
SPECT: single-photon emission computed tomography
SPIRIT: Standard Protocol Items: Recommendations for Interventional Trials
UCLA: University of California, Los Angeles
UMIN: University Hospital Medical Information Network
VR: virtual reality
