# Supplementary material for: Effectiveness of a Virtual Reality Open-Air Bath Program in Reducing Loneliness and Improving Brain Function for Dementia Prevention in Older Adults: Protocol for a Prospective Randomized Crossover Study
Source: JMIR Res Protoc. 2024 Aug 1;13:e57101. doi: 10.2196/57101 (PMC11327636; doi:10.2196/57101)
Supplement: Multimedia Appendix 2 [file resprot_v13i1e57101_app2.pdf]

December 15, 2023 ver.3

Agreement

To principal investigator

Affiliation / Position Professor, Department of Psychiatry, Graduate School of Medicine, Kyoto Prefectural University of Medicine

Name: Mr. Jin Narimoto

I, (name.   ), have fully understood the significance, purpose, methods, and method of protecting my personal information of the research plan and agree to participate in the research plan. I fully understand the significance, purpose, methods, and protection of personal information of the research plan, and I agree to participate in the plan.

1 Items explained and understood (Please tick the appropriate box)

☐ Purpose and Significance of the Plan
☐ Method and duration of the study
☐ Reason for selection as a research subject
☐ Burden incurred and expected risks and benefits
☐ Compensation for health damage
☐ Participation in the plan should be voluntary.
☐ The ability to withdraw at any time, even if you have agreed to participate.
☐ Disclosure of information on research
☐ Handling of Personal Information
☐ Methods of preservation and destruction of samples and information
☐ Research Funding and Conflicts of Interest
☐ Handling of research results
☐ Financial burden or gratuities

2 Consent to Research Cooperation

(1) I agree that the sample/information I provide will be used for this research.

☐ Yes ☐ No

*If you answered "yes" to question (1), please proceed to question (2) and mark either ✓.

(2) I agree that the sample/information I provide will be used for this research and will be stored for a long period of time, and will be used in the future for newly planned and conducted research, subject to approval by the University's Medical Ethics Review Committee.

*If "No" is selected, the samples and information will be disposed of immediately after the completion of this research.

☐Yes ☐ No

Date.________________

Research participant's name

*Name and seal or self-signature

Date of Birth

Address

Phone number

Consent Withdrawal Letter

To principal investigator

Affiliation / Position Professor, Department of Psychiatry, Graduate School of Medicine, Kyoto Prefectural University of Medicine

Name: Mr. Jin Narimoto

I, (name ____________), withdraw my consent to participate in the study entitled Virtual Reality program to reduce loneliness and social isolation.

Date._________________

Research participant's name *Name and seal or self-signature

Date of Birth

Address

Phone number
